# Supplementary material for: Use of gentamicin-collagen sponge (Collatamp® G) in minimally invasive colorectal cancer surgery: A propensity score-matched study
Source: PLoS One. 2022 Mar 28;17(3):e0264513. doi: 10.1371/journal.pone.0264513 (PMC8959166; doi:10.1371/journal.pone.0264513)
Supplement: Attachment — Submitted filename: Response to the reviewers_final.docx [file pone.0264513.s001.docx]

Reviewer #1: Major Compulsory Revisions:

1. Query: Authors should mention in the limitations as the inadequate sample size might lead to min-interpretation of results.

Response: We appreciate the reviewer’s thoughtful comments. Accordingly, we have presented this as a limitation

2. Conclusion paragraph: We demonstrated that, in patients with colon or rectal cancer who undergo laparoscopic resection, applying a gentamicin-collage sponge to the mini-laparotomy wound did not reduce the frequency of SSIs, even in high-risk patients.
Authors were suggested to correct "who undergo laparoscopic resection" to "who undergo minimally invasive surgery".

Response: Accordingly, we have revised the manuscript to reflect your suggestion.
